# Reduced DNA methylation patterning and transcriptional connectivity define human skin aging

**DOI:** 10.1111/acel.12470

**Published:** 2016-03-23

**Authors:** Felix Bormann, Manuel Rodríguez‐Paredes, Sabine Hagemann, Himanshu Manchanda, Boris Kristof, Julian Gutekunst, Günter Raddatz, Rainer Haas, Lara Terstegen, Horst Wenck, Lars Kaderali, Marc Winnefeld, Frank Lyko

**Affiliations:** ^1^ Division of EpigeneticsDKFZ‐ZMBH AllianceGerman Cancer Research CenterHeidelbergGermany; ^2^ University Tumor Center DüsseldorfUniversity of DüsseldorfDüsseldorfGermany; ^3^ Research and DevelopmentBeiersdorf AGHamburgGermany; ^4^ Institute for BioinformaticsUniversity Medicine GreifswaldGreifswaldGermany; ^5^ Present address: Department of BiotechnologyBeuth University of Applied SciencesBerlinGermany

**Keywords:** age prediction, DNA methylation, epidermis, epigenetic drift, epigenetics, skin aging

## Abstract

Epigenetic changes represent an attractive mechanism for understanding the phenotypic changes associated with human aging. Age‐related changes in DNA methylation at the genome scale have been termed ‘epigenetic drift’, but the defining features of this phenomenon remain to be established. Human epidermis represents an excellent model for understanding age‐related epigenetic changes because of its substantial cell‐type homogeneity and its well‐known age‐related phenotype. We have now generated and analyzed the currently largest set of human epidermis methylomes (*N* = 108) using array‐based profiling of 450 000 methylation marks in various age groups. Data analysis confirmed that age‐related methylation differences are locally restricted and characterized by relatively small effect sizes. Nevertheless, methylation data could be used to predict the chronological age of sample donors with high accuracy. We also identified discontinuous methylation changes as a novel feature of the aging methylome. Finally, our analysis uncovered an age‐related erosion of DNA methylation patterns that is characterized by a reduced dynamic range and increased heterogeneity of global methylation patterns. These changes in methylation variability were accompanied by a reduced connectivity of transcriptional networks. Our findings thus define the loss of epigenetic regulatory fidelity as a key feature of the aging epigenome.

## Introduction

DNA methylation represents a conserved and highly dynamic epigenetic modification of animal and plant genomes (Law & Jacobsen, 2010). In the human genome, about 4% of the cytosines are modified by methylation. A key feature is the high specificity for CpG dinucleotides, which ensures the heritability of methylation patterns through a semiconservative maintenance mechanism (Jones, 2012). DNA methylation of gene regulatory elements, such as promoters and enhancers, is generally considered to be incompatible with activated gene expression (Schubeler, 2015). However, promoters and enhancers represent only a very minor part of the methylated genome, and the global correlation between DNA methylation and gene expression patterns remains to be fully understood.

Over the past few years, several landmark studies have shown that mammalian development and cellular differentiation are characterized by widespread changes of genomic DNA methylation patterns (Smith & Meissner, 2013). This has reinforced the notion that mammalian DNA methylation primarily functions as an epigenetic mark for cell‐type specification (Roadmap Epigenomics Consortium *et al*. 2015). Furthermore, DNA methylation changes are also considered to facilitate cellular adaptation to changing environments and have repeatedly been linked to human diseases and aging (Feinberg, 2007). While the functional role of epigenetic mechanisms in aging remains to be fully understood, altered DNA methylation and chromatin remodeling represent key elements of the aging process in a large number of model systems (Fraga & Esteller, 2007; Weidner & Wagner, 2014; Benayoun *et al*., 2015).

Several studies have also described age‐related DNA methylation changes in human tissues, a phenomenon that has been termed ‘epigenetic drift’ (Feil & Fraga, 2012; Teschendorff *et al*., 2013). While the key features of epigenetic drift remain to be defined, several observations have been made that characterize age‐related methylation changes across human tissues. These include the hypermethylation of stem cell genes (Rakyan *et al*., 2010; Teschendorff *et al*., 2010), a general linear correlation between the methylation level of certain CpGs and the chronological age (Hannum *et al*., 2013; Horvath, 2013; Weidner *et al*., 2014), and the hypermethylation of CpG islands (Yuan *et al*., 2015). The molecular and phenotypic consequences of these alterations remain a topic of active research.

The aging phenotype of human skin is well‐documented and of considerable medical and economical importance. The epidermis is directly exposed to the environment and may therefore contain particularly pronounced environmental imprints on the epigenetic level. Skin samples can be obtained from healthy volunteers over a broad age range by well‐accepted and tolerable procedures. These samples are characterized by a high degree of cell‐type homogeneity (Gunin *et al*., 2011), thus mitigating the confounding effects of age‐related changes in the cellular composition of many human tissues, such as blood (Houseman *et al*., 2012; Lowe & Rakyan, 2014). The combination of these factors establishes human skin as a particularly useful model for the analysis of age‐related epigenetic changes.

Recent technological advances support the generation of genomewide DNA methylation profiles and greatly facilitate our understanding of epigenetic modification patterns. This is exemplified by whole‐genome bisulfite sequencing, a method that allows the methylation analysis of complete genomes at single‐base resolution (Lister & Ecker, 2009). However, whole‐genome bisulfite sequencing is expensive and time‐consuming and requires substantial computational resources. Furthermore, methylation levels are often similar between neighboring CpGs, and only a minor fraction of the mammalian genome undergoes dynamic methylation changes (Ziller *et al*., 2013). This suggests that a subset of CpG dinucleotides can be used to analyze genomic DNA methylation patterns (Ziller *et al*., 2013). In this context, the Infinium 450k array represents the most widely used platform and allows the methylation analysis of more than 450 000 cytosine residues in the human genome. The corresponding probes have been selected to cover both general and specific features that are commonly linked to epigenetic gene regulation (Bibikova *et al*., 2011).

Previous studies that investigated DNA methylation changes associated with human skin aging were characterized by several important limitations. Our first study utilized an earlier version of the methylation array that was limited to 27 000 CpG dinucleotides with a strong bias for promoter regions (Gronniger *et al*., 2010). Our second study used whole‐genome bisulfite sequencing, but provided information only for single pools of five young and old epidermis samples, respectively (Raddatz *et al*., 2013). A more recent study used a combination of 450k methylation arrays and low‐coverage bisulfite sequencing, but focused mostly on the accumulated effects of sun exposure in aged skin (Vandiver *et al*., 2015). As such, the general epigenetic changes associated with intrinsic skin aging and their relationships to age‐related epigenetic changes described in other tissues remained to be analyzed. We have now used 450k methylation arrays to investigate the epidermal methylation patterns of 108 donors from a wide range of age groups. A detailed analysis of the resulting data identified key features of the age‐related epigenetic drift.

## Results

### General features of the aging skin methylome

Previous analyses of methylation changes associated with skin aging were limited by low methylation coverage, low sample numbers and/or heterogeneous donor groups. For our initial analysis, we therefore obtained epidermis samples from 24 young (18–27 years) and 24 old (61–78 years) volunteers (Fig. S1). To reduce the effects of confounding factors, all samples were obtained by the same procedure (punch biopsies), from the same location (outer forearm) and from a donor group with a defined sex (female), ethnicity (Caucasian) and health status (disease‐free). DNA was purified from all samples in parallel and subjected to Infinium 450k methylation profiling. All resulting datasets passed stringent quality controls and were subsequently analyzed using state‐of‐the‐art statistical tools.

**Figure 1:**
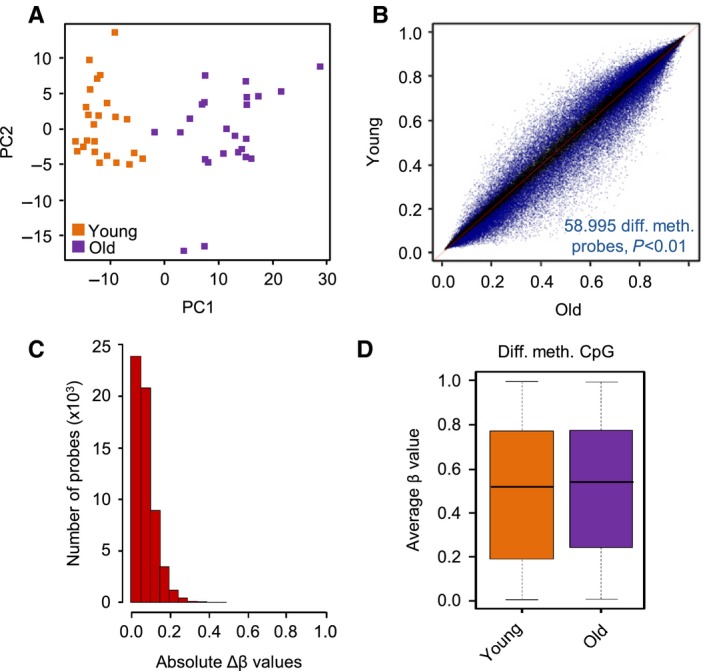
Age‐related methylation differences are characterized by relatively small effect sizes. (A) Principal component analysis of epidermis methylomes clearly separates young and old samples. (B) Scatter plot comparing the epidermis methylomes of 24 young (18–27 years) and 24 old (61–78 years) volunteers. A total of 58 995 differentially (adjusted *P* < 0.01) methylated CpG probes are indicated in blue. (C) Size distribution of methylation differences. For most of the 58 995 differentially methylated probes, this difference is < 0.2. (D) Box plot showing a slight global DNA hypermethylation in the epidermis of old volunteers.

Principal component analysis on all 450k probes achieved a clear separation of young and old samples (Fig. 1A). Indeed, singular value decomposition (SVD) analysis of β values identified age as the major source of variation in our datasets (Fig. S2). A comparison of the methylation patterns of the young and old sample groups using minfi (Aryee *et al*., 2014) revealed 58 995 probes with a statistically significant (adjusted *P* < 0.01) methylation change (Figs 1B and S3). Further analysis revealed that the large majority of differentially methylated probes showed only minor (Δβ < 0.1) quantitative methylation differences (Fig. 1C). Also, average β values of the differentially methylated probes showed only a very slight trend toward hypermethylation in old samples (Fig. 1D), but failed to indicate any large‐scale methylation changes, such as an age‐related global loss of methylation marks (Heyn *et al*., 2012; Vandiver *et al*., 2015). Finally, we also analyzed the data from 980 probes in non‐CpG sequence context. In agreement with recently published data (Schultz *et al*., 2015), we detected low, but significant levels of non‐CpG methylation in the human epidermis (Fig. S4). Differences between young and old samples appeared minor (Fig. S4), with only three isolated probes achieving statistical significance (data not shown). Together, these results suggest a general stability of the human epidermis methylome during aging and indicate the presence of locally restricted age‐related methylation differences.

**Figure 2:**
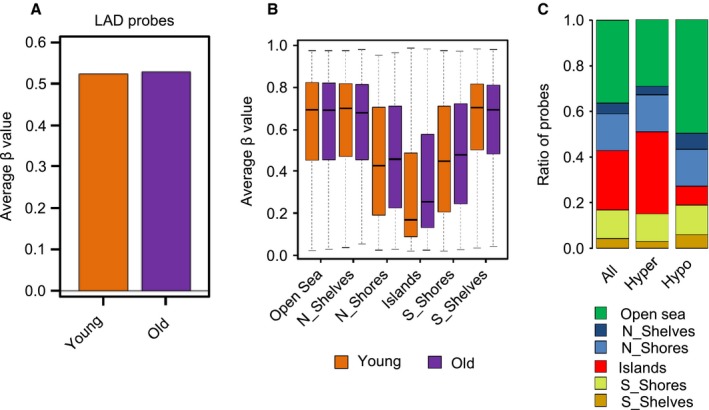
Age‐related methylation changes are locally restricted. (A) Average β values of probes assigned to lamina‐associated domains. Orange bar: young samples, purple bar: old samples. (B) Methylation status of different epigenomic substructures in the epidermis of young and old volunteers. The box plots show a highly significant (*P* = 2.0e‐17) hypermethylation of CpG islands in old donors. (C) Fractions of hyper‐ and hypomethylated CpGs within different epigenomic substructures. The graph shows a significant enrichment of CpG island‐associated probes among the hypermethylated CpGs, and a concomitant decrease of CpG island‐associated probes among the hypomethylated CpGs.

Both hypomethylation and hypermethylation have previously been implied in human tissue aging (Rakyan *et al*., 2010; Teschendorff *et al*., 2010; Heyn *et al*., 2012; Vandiver *et al*., 2015; Yuan *et al*., 2015). This includes the hypomethylation of megabase‐scale blocks, which has recently been described in aged and sun‐exposed human skin (Vandiver *et al*., 2015). We used the same (Vandiver *et al*., 2015) approaches to identify hypomethylated blocks in our datasets. Surprisingly, however, none of the identified blocks showed an average methylation difference of > 0.1 in our dataset (see Fig. S5 for an example). We therefore used an alternative approach and defined hypomethylated blocks through their association with lamina‐associated domains (LADs, Guelen *et al*., 2008). However, we could not observe an age‐related decrease in the average methylation level of LAD‐associated probes in our dataset (Figs 2A and S5). These findings strongly suggest that hypomethylated blocks are not a major feature of intrinsic skin aging.

We also used minfi to analyze methylation patterns at the level of epigenomic substructures, such as CpG islands, shores, shelves, and open sea regions (Irizarry *et al*., 2009; Sandoval *et al*., 2011). The results again indicated only minor differences between the young and old samples for most substructures, but revealed a robust and highly significant (*P* = 2.0e‐171) hypermethylation of CpG islands in the old samples (Fig. 2B). Similarly, CpG island‐associated probes were distinctly overrepresented among hypermethylated probes and underrepresented among hypomethylated probes (Fig. 2C). Hypermethylation of CpG islands represents a conserved feature of aging human cells (Yuan *et al*., 2015).

### Gradual methylation changes associated with skin aging

To confirm and further characterize these methylation differences, we obtained epidermis samples from an additional group of 60 healthy female volunteers that also included intermediate ages (Fig. S1). We generated Infinium 450k methylation profiles from all samples and integrated the resulting data into our existing dataset. Hierarchical clustering on the complete dataset failed to reveal any batch effects (Fig. S6) and thus allowed the use of a merged dataset of 108 samples for the subsequent analysis.

**Figure 3:**
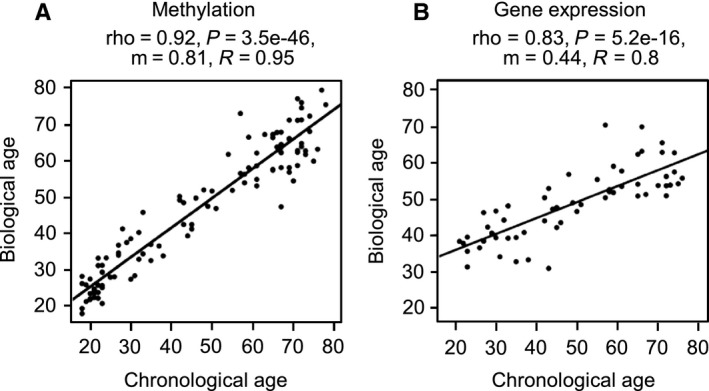
Continuous methylation changes predict chronological age. Biological age predicted from the methylation (left, *N* = 108) or gene expression (right, *N* = 59) profiles is plotted over the chronological age of the samples. Predictions were made using a support vector machine, using leave‐one‐out cross‐validation and using all available probes on the respective platform.

When we used the merged dataset (*N* = 108) to predict the age of our samples using a published predictor based on a set of 353 tissue‐independent probes (Horvath, 2013), we obtained a good correlation between chronological and predicted biological age (ρ = 0.91, *P* < 2.2e‐16). However, when calculating the linear regression of the data, the regression curve showed a slope of only 0.55. Furthermore, the average absolute prediction error was 14.5 years (Fig. S7), clearly showing that the published predictor underestimates the true age of epidermis samples. We therefore used a linear support vector machine (SVM) to predict biological age based on our skin methylation profiles. SVM training was performed with leave‐one‐out and also 10‐fold cross‐validation using the complete set of 450k probes as independent variables, and chronological age as dependent variable. We then compared the predicted age for each of the 108 samples with the chronological age. This resulted in a very high correlation (ρ = 0.92, *P* < 2.2e‐16) between estimated biological and chronological age, comparable to the published predictor, but with an average absolute prediction error of less than 5.25 years (Fig. 3A), thus clearly confirming gradual age‐related methylation changes in the human epidermis. We furthermore validated our calibrated predictor on the *N* = 18 sun‐exposed epidermis samples from the (Vandiver *et al*., 2015) study, resulting in very high correlation between predicted and true age (ρ = 0.96, *P* = 1.66e‐10, *R*‐squared = 0.93) and an average absolute prediction error of 6.72 years (Fig. S7). On the same dataset, the published method (Horvath, 2013) achieved slightly inferior results in terms of correlation (ρ = 0.89, *P* = 7.6e‐7, *R*‐squared = 0.794), but again underestimated the biological age with an average absolute prediction error of 12.1 years (Fig. S7). Finally, we compared predictions using methylation data with a linear SVM that had been trained similarly on gene expression data (*N* = 59). As gene expression profiles were available for only 59 samples, we also performed predictions on the methylation data by (repeated) random subsampling of *N* = 59 methylation profiles. While we still achieved an average absolute error of 6.48 years on the subsampled methylation data (representative run: ρ = 0.938, *P* < 2.2e‐16), the prediction accuracy on the gene expression data was significantly worse (correlation ρ = 0.83, *P* = 5.2e‐16), with an average absolute prediction error of 9.48 years (Fig. 3B). The substantially higher accuracy of methylation‐based age prediction further illustrates the importance of age‐related methylation changes.

### Discontinuous age‐related methylation changes

**Figure 4:**
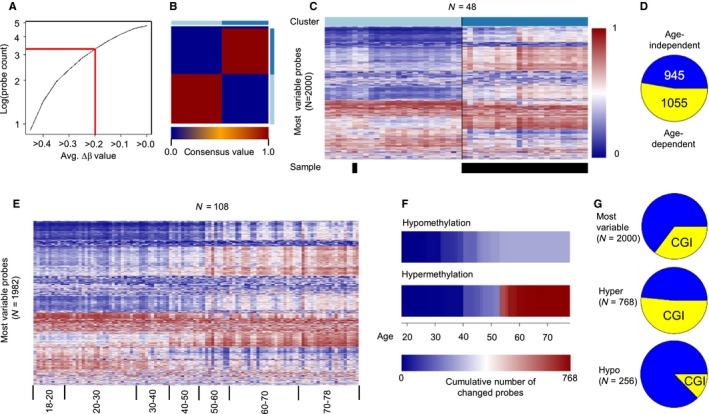
Discontinuous methylation changes during aging. (A) Number of differentially methylated probes in relation to their age‐related (young vs. old) average methylation difference. Approximately 2000 probes showed an average age‐related methylation difference of greater than or equal to 0.2. (B) Consensus Matrix for two cluster centers after consensus clustering of the 2000 most variable probes (young vs. old). Only consensus values of 0 (two samples never cluster together) or 1 (two samples always cluster together) were observed, indicating optimum clustering into two groups. (C) β value heatmap of the 2000 most variable probes. β values were colored from blue (β = 0) to red (β = 1). Colors in the bar above the matrix indicate cluster assignment. Epidermis methylomes from the old sample group are indicated by black boxes below the heatmap. Only one sample appeared in the wrong cluster. (D) Distribution of age‐dependent and age‐independent β values within the 2000 most variable probes. Yellow: age‐dependent probes; Blue: age‐independent probes. (E) β value heatmap of the most variable probes within the complete dataset after sorting by age. Discontinuous methylation changes occur for a subset of probes between the ages of 40 and 60. (F) Identification of probes showing discontinuous methylation changes by recursive partitioning. The heatmaps represent the cumulative number of probes showing a discontinuous β value change at the specified age. Hypomethylation occurred in less probes than hypermethylation and at different ages. (G) Fraction of CpG island‐associated probes (yellow) among the most variable (top), hypermethylated (middle) and hypomethylated (bottom) probes.

To further investigate age‐related methylation changes, we then focused our analyses on the 2000 most variably methylated probes. This number was chosen because an initial analysis of our young/old (*N* = 48 dataset) revealed that 1918 probes showed an average methylation difference of Δβ ≥ 0.2, which is often used to determine ‘robust’ methylation changes (Fig. 4A). A consensus cluster analysis of these 2000 probes identified two clearly defined clusters consisting of 25 and 23 samples, respectively (Fig. 4B). With a single exception, these consensus clusters correctly identified the young and old sample groups (Fig. 4C). Hierarchical clustering of the β values from both clusters (Fig. 4C) also showed two distinct patterns of variation among the 2000 probes: While 945 (47%) probes showed age‐independent interindividual variation, 1055 (53%) probes showed consistent age‐related differences (Fig. 4D), which confirms the presence of specific age‐related methylation changes among the most variable probes.

Interestingly, a closer examination of the 2000 most variable probes in the complete (*N* = 108) dataset strongly suggested that a substantial fraction of age‐related methylation changes accumulated during narrowly defined windows (Fig. 4E). The corresponding time points were subsequently identified by recursive partitioning for each of the 2000 probes. This identified step‐wise hypomethylation for 256 probes and step‐wise hypermethylation for 768 probes (Figs 4F and S8). Hypomethylation appeared evenly distributed between ages 30 and 50, while hypermethylation accumulated in two pronounced peaks around 40 and 55 years, respectively (Figs 4F and S8). Because CpG island hypermethylation is a conserved feature of aging human cells, we also tested whether discontinuous methylation changes are enriched in this genomic feature. Indeed, out of the 768 probes defining discontinuous hypermethylation events, 396 (52%) were located in CpG islands, while only 13% (32 of 256) of the discontinuously hypomethylated probes were located in CpG islands (Figs 4G and S8B). Finally, while differences in sample identity and age distribution preclude a direct validation, overall similar changes could be observed with published data from an independent study (Fig. S8 (Vandiver *et al*., 2015)). Together, these findings identify discontinuous methylation changes as a novel feature associated with human skin aging.

### Age‐related erosion of epigenetic regulation

**Figure 5:**
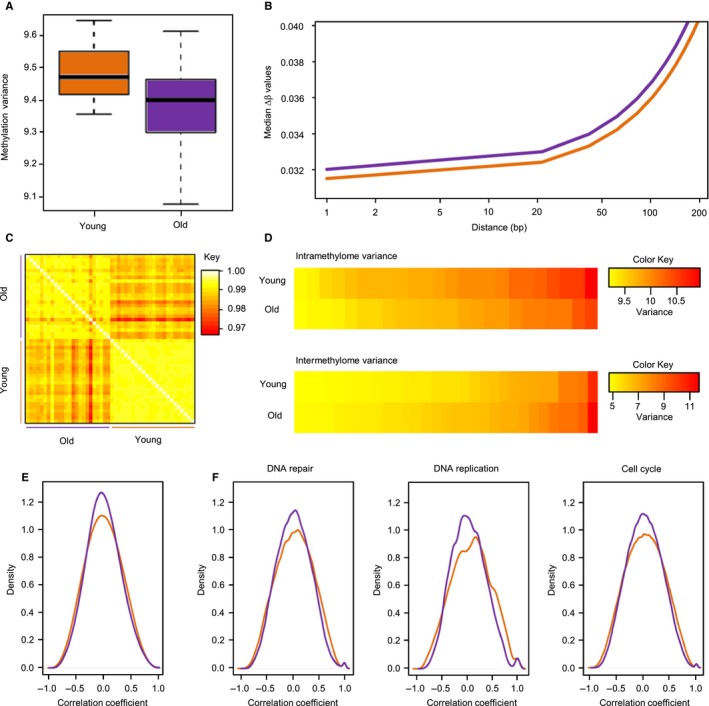
Erosion of methylation patterns in old samples. (A) Intramethylome variance for young and old samples. (B) Spatial correlation of methylation marks. Lines indicate smoothened medians of distance‐dependent β value differences for the young (orange) and old (purple) datasets. (C) Methylation heterogeneity analysis. Pearson correlation coefficients were calculated after performing a probe‐by‐probe β value comparison for all young and old samples. (D) Heatmaps of intramethylome and intermethylome variance. For the intermethylome variance, results are shown for the 30 most variable probes. (E) Global pairwise correlation of gene expression data from young (orange) and old (purple) samples. The plot represents the overall density distribution of the correlation coefficient of genes. (F) Correlation density plots for specific pathways from the Reactome database. Young and old samples are shown in orange and purple, respectively.

To further characterize the global features of young and old epidermis methylomes, we compared the within‐sample methylation variance for all 450k probes (see Experimental procedures for details). We observed a highly significant (*P* = 0.0016) difference between the old and young groups, with the young samples showing an overall higher variability in their methylation profiles (Fig. 5A). These results indicate that the dynamic range of skin methylomes becomes reduced with age. To corroborate our finding, we analyzed the spatial correlation in the methylation levels of neighboring CpGs, which has been shown to become reduced in T cells from old human donors (Heyn *et al*., 2012). In agreement with earlier findings in T cells (Heyn *et al*., 2012), our results show that the spatial correlation of the methylation levels of neighboring CpGs is slightly but significantly (*P* = 5.4e‐07) reduced in older donors (Fig. 5B), which is again consistent with an age‐related reduction in methylation patterning. Finally, when we calculated average Pearson correlation coefficients for probe‐by‐probe comparisons in our datasets, methylation patterns appeared highly homogeneous among the young epidermis samples (Fig. 5C). Notably, correlation coefficients became distinctly reduced in old samples and were also reduced when old samples were compared to other old samples (Fig. 5C). These effects were further confirmed when the β value variances were calculated individually for each sample and for each probe per sample group to determine intra‐ and intermethylome variance, respectively (Fig. 5D). These findings illustrate how skin methylomes erode with age and identify a novel and important feature of the age‐related epigenetic drift.

We next addressed the question whether decreasing variability was also observable at the level of gene expression, but could not observe a similar global trend (data not shown). However, it has been described that aging mouse tissues have a decreased correlation of gene expression (Southworth *et al*., 2009). We therefore computed pairwise Pearson correlation coefficients between the 30% most variable genes in the young and old sample groups (see Experimental procedures for details). The results showed that the number of gene pairs with no expression correlation was distinctly increased in the old samples (Fig. 5E). We also performed a similar correlation analysis for specific gene networks using selected categories from the Reactome database. Our results again showed a distinct age‐related loss of correlated gene expression in these networks (Fig. 5F), consistent with the transcriptome‐wide result. Together, these findings support the notion that aging is accompanied by a reduced fidelity of epigenetic regulation in the human epidermis.

## Discussion

Epigenetic drift has been suggested to be a major hallmark of aging human tissues (Feil & Fraga, 2012; Teschendorff *et al*., 2013). However, while numerous studies have described age‐related methylation changes, the specific characteristics of this phenomenon have not been defined yet. We have used human skin as a paradigm for tissue aging because its well‐known aging phenotype and its high degree of cell‐type homogeneity during adult age (Gunin *et al*., 2011), which mitigates major confounding effects caused by age‐related changes in cell‐type proportions (Houseman *et al*., 2012; Lowe & Rakyan, 2014). In agreement with our previous studies that were carried out either at lower resolution or with smaller sample sizes (Gronniger *et al*., 2010; Raddatz *et al*., 2013), we find that age‐related methylation changes appear rather moderate and do not compromise the overall integrity of the epidermis methylome. Nevertheless, we identified a variety of specific age‐related methylation changes. In contrast to prior work by others (Hannum *et al*., 2013; Horvath *et al*., 2013), where whole‐blood samples and different tissues were used to develop a predictive signature of biological age, we achieved significantly improved prediction accuracy by training the prediction algorithm on epidermis samples.

In agreement with previous analyses (Yuan *et al*., 2015), we observed a significant age‐related hypermethylation of CpG island‐associated probes. Interestingly, this effect was strongly enriched during two specific age windows, at 40–45 and 50–55 years. Considering that our samples were exclusively derived from female volunteers, it seems reasonable to link the latter window to menopause, which is also known to distinctly accelerate skin aging (Thornton, 2013). The high temporal and spatial specificity of these methylation changes suggests that defined signaling pathways, such as estrogen signaling (Emmerson & Hardman, 2012), may be involved in their establishment.

**Figure 6:**
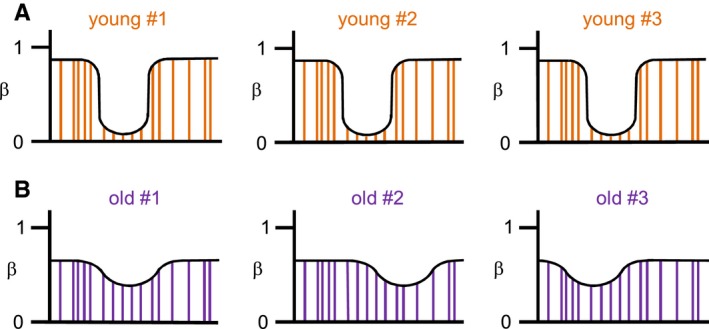
Model of age‐related changes in methylation patterning. Panels illustrate standard human methylation patterns with alternating regions of highly and lowly methylated CpGs (vertical lines). (A) Young epidermis samples display methylomes with a high dynamic range (high intramethylome variance) and a low intermethylome variance. (B) In contrast, old samples show methylomes with a reduced dynamic range (resulting in a lower intramethylome variance) and a higher intermethylome variance.

Our results also describe an age‐related erosion of DNA methylation patterns that is characterized by two distinct features: (i) While the topology of young methylomes is characterized by sharply demarcated regions of (almost) complete and (almost) absent methylation, old methylomes appeared to be less clearly defined, which is reflected by the significantly reduced variance and spatial correlation within methylomes. (ii) While young methylomes are highly similar among each other, old methylomes appeared to be substantially more heterogeneous. Hence, while methylation patterning within an individual becomes more homogeneous with age, the differences between individuals increase (Fig. 6). These findings define important features of the epigenetic drift associated with aging. Interestingly, methylation heterogeneity has been observed during the long‐term culture of human fibroblasts (Landan *et al*., 2012) and in aging human mesenchymal stem cells (Fernandez *et al*., 2015). Furthermore, increased methylation heterogeneity has also been associated with cellular senescence in cultured human epithelial cells (Lowe *et al*., 2015).

The effects of age‐related methylation changes on gene expression patterns have been analyzed in several previous studies. Somewhat surprisingly, however, no global correlations could be established and methylation‐related expression changes generally appeared very limited (Heyn *et al*., 2012; Raddatz *et al*., 2013; Reynolds *et al*., 2014; Zykovich *et al*., 2014; Yuan *et al*., 2015). These findings support the notion that age‐related methylation changes function to stabilize pre‐existing gene expression patterns (Yuan *et al*., 2015). Alternatively, age‐related gene expression changes might also be too subtle to achieve statistical significance in classical differential expression analyses. The analysis of gene co‐expression networks provides an opportunity to analyze transcriptional deregulation at a higher level of complexity (Southworth *et al*., 2009), and our findings demonstrate a reduced connectivity of gene expression in old samples. These results are in agreement with earlier findings in aging mice (Southworth *et al*., 2009) and suggest that the age‐related erosion of methylation patterns is accompanied by a reduced fine‐tuning in the transcriptional circuitry, possibly through methylation‐dependent changes in transcription factor binding (Yuan *et al*., 2015).

It will be particularly interesting to identify the factors that are involved in the age‐related erosion of DNA methylation patterns. For example, it has been shown that the Dnmt3a DNA methyltransferase plays an important role in shaping the topology of DNA methylation patterns. When Dnmt3a was deleted in mouse hematopoietic stem cells, DNA methylation canyons became eroded (Jeong *et al*., 2014), causing reduced methylation variance and reduced spatial correlation of methylation marks at specific target loci. Similar observations were made in mouse embryonic fibroblasts that lack the Tet1 and Tet2 dioxygenases (Wiehle *et al*., 2016). Interestingly, we also observed a significant relationship between age and the normalized gene expression level of *TET1*, which becomes moderately, but significantly (*P* < 0.05) reduced with age (Fig. S9). High‐resolution methylation mapping of genetically engineered cells will be required for a detailed characterization of the mechanisms that are involved in this process.

## Experimental procedures

### Samples

Epidermis samples for the first (*N* = 48) dataset were isolated from punch biopsies that had been obtained from the outer forearm of 24 young (18–27 years) and 24 old (61–78 years) volunteers. Epidermis samples for the second (*N* = 60) dataset were obtained as suction blister roofs from the outer forearm of 60 volunteers aged 20–79 years (Skin Investigation And Technology, Hamburg, Germany). All volunteers shared the same gender (female), ethnicity (Caucasian) and health status (disease‐free). All studies were approved by the Ethics Committee of the Medical Association of Hamburg (PV 3107, PV 4280) and by the Freiburg Ethics Commission International (011/1973), and were performed in agreement with the recommendations of the Declaration of Helsinki.

### Analysis of Infinium 450k data

Preprocessing of raw data.idat files (read‐in, quality control, SWAN) from Infinium 450k microarrays was performed using the minfi package (Aryee *et al*., 2014). Probes located on the sex chromosomes, or which failed detection *P*‐value testing in at least one sample, were removed from the analysis. Likewise, probes showing cross‐reactivity to at least one distant site, and thus allowing a mismatch of one base in the probe sequence, were eliminated. Finally, probes containing a SNP with an allele frequency of at least 0.01, and located a maximum of five bases away from the single‐base extension (SBE) site, were also removed. β values and M values were extracted from the remaining probes. Prediction of batch effects was carried out by singular value decomposition (SVD) using the champ bioconductor package (Morris *et al*., 2014). Identification of differentially methylated CpG probes between sample groups was performed using a linear model fit of the M values with subsequent empirical Bayes reduction in standard errors from the estimated methylation differences, as described in the limma r package (Ritchie *et al*., 2015). As the distribution of unadjusted *P*‐values showed no influence of potential confounding factors (Fig. S3), raw *P*‐values were adjusted using an FDR method (Benjamini–Hochberg).

### Identification of large hypomethylated blocks

To identify DNA hypomethylation blocks, the blockFinder algorithm of the minfi package was used as described previously (Aryee *et al*., 2014). Block finding was performed on open sea probes using the default parameters and a B parameter of 100. Blocks were filtered for an adjusted *P*‐value < 0.05 (family‐wise error rate) and an overall absolute average methylation difference > 0.1. For visualization, β values of the probes within the identified blocks were averaged for young and old epidermis datasets and smoothed using a locally weighted regression approach. LAD association of Infinium 450k probes was determined using published datasets (Guelen *et al*., 2008).

### Consensus clustering

To identify distinct age‐dependent methylation clusters, consensus clustering was performed using the β values of the 2000 most variable probes from the 24 old vs. 24 young epidermis dataset (i.e., the probes with the highest standard deviations). The consensus cluster algorithm is included in the ConsensusClusterPlus R package (Wilkerson & Hayes, 2010) and was applied using the k‐means cluster algorithm, 1000 bootstraps, an item subsampling proportion of 0.8, and a feature subsampling proportion of 1. For visualization, all samples per cluster, as well as the 2000 probes, were further ordered by hierarchical clustering.

### Identification of discontinuous methylation changes

The β values of the 2000 most variable probes were re‐calculated using the complete set of samples (*N* = 108). After quality control, 1982 probes were considered further. Probes were defined as age‐dependent if there were no overlaps in the average ± standard deviation intervals between both datasets. For the identification of discontinuous methylation changes, β values were plotted with respect to the age and subjected to a recursive partitioning algorithm implemented in the rpart r package. Assuming only one age‐dependent methylation change, the maximum depth parameter was set to 1. Further analysis was restricted to probes showing a relative error ≤ 0.75 after recursive partitioning. Probes were subsequently assigned to one of five groups based on the left branch value identified by recursive partitioning. The resulting probe sets were then further stratified using the direction and the calculated age of the methylation change.

### Methylome variance analysis

The young and old datasets were tested for intermethylome and intramethylome variance by calculating either the M value variance of all probes within the old or the young epidermis datasets, or by calculating the M value variance of all samples within the old and the young epidermis datasets. For the spatial correlation analysis, all pairs of probes present in the 450k microarray and located within a distance of maximum 1000 bases were identified. Then, after obtaining the average β values of all these probes in the old and young datasets, their absolute difference (Δβ value) was calculated for each pair of probes. Finally, the median of all Δβ values per distance was calculated, smoothened and plotted for both young and old epidermis data.

### Gene expression analysis

mRNA expression profiles (*N* = 59) were generated using Agilent SurePrint G3 arrays from the same set of epidermis samples (outer forearm suction blister roofs) that were also used for methylation analyses. To determine the correlation of gene expression patterns, we assigned young (20–30 years, *N* = 12) and old (> 60 years, *N* = 19) samples to two distinct groups. Due to the large number of pairwise correlations between all genes on the array (≈30 000 × 30 000 = 900 000 000 pairwise correlation coefficients), we restricted the analysis to the top 30% genes with the highest variability between samples and then computed pairwise Pearson correlation coefficients between all these genes in the old sample group and in the young sample group, respectively.

## Data access

All array data have been deposited in the ArrayExpress database under the accession numbers E‐MTAB‐4385 (methylation data) and E‐MTAB‐4382 (expression data).

## Funding

This work was supported by the German Cancer Research Center and Beiersdorf AG.

## Conflict of interest

SH, BK, LT, HW, and MW are employees of Beiersdorf AG. LK and FL received consultation fees from Beiersdorf AG.

## Author contributions

FB, MRP, HM, JG, GR, and LK analyzed the data. SH, BK, LT, HW, and MW organized the collection of samples. RH contributed to the design of the data analysis pipeline. SH, MW, and FL conceived the study. FL wrote the study with contributions from FB, MRP, and LK. All authors read and approved the final manuscript.

## Supporting information


**Fig. S1** Sample sets used in this study.
**Fig. S2** Singular Value Decomposition (SVD) analysis of β values.
**Fig. S3** *P*‐value frequency histogram.
**Fig. S4** Analysis of non‐CpG methylation.
**Fig. S5** Analysis of hypomethylated blocks.
**Fig. S6** Cluster analysis of the complete (*N* = 108) sample set.
**Fig. S7** Application of age prediction methods on our data and on a published dataset.
**Fig. S8** Identification of discontinuous methylation changes by recursive partitioning.
**Fig. S9** Correlation between age and the normalized gene expression level of *TET1*.
