# Supplementary material for: Reduced DNA methylation patterning and transcriptional connectivity define human skin aging
Source: Aging Cell. 2016 Mar 23;15(3):563–71. doi: 10.1111/acel.12470 (PMC4854925; doi:10.1111/acel.12470)
Supplement: Supplementary file 1 — Fig. S1 Sample sets used in this study. Fig. S2 Singular Value Decomposition (SVD) analysis of β values. Fig. S3 P‐value frequency histogram. Fig. S4 Analysis of non‐CpG methylation. Fig. S5 Analysis of hypomethylated blocks. Fig. S6 Cluster analysis of the complete (N = 108) sample set. Fig. S7 Application of age prediction methods on our data and on a published dataset. Fig. S8 Identification of discontinuous methylation changes by recursive partitioning. Fig. S9 Correlation between age and the normalized gene expression level of TET1. [file ACEL-15-563-s001.docx]

**Supporting Information**

**Reduced DNA methylation patterning and transcriptional connectivity**

**define human skin aging**

Contents:

Figures S1-S9

**
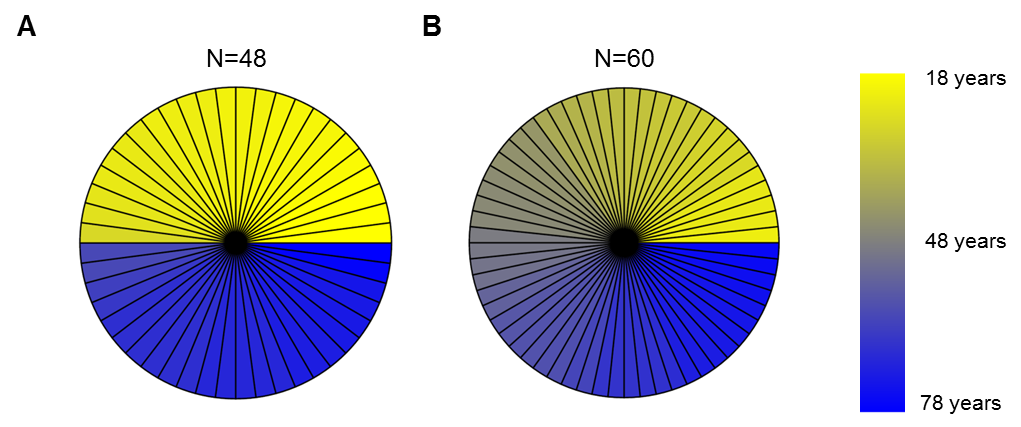
**

**Fig. S1** Sample sets used in this study. (A) 24 young and 24 old epidermis samples. (B) Additional 60 epidermis samples. The corresponding ages of the donors are indicated for each sample set, ranging from the youngest (yellow) to the oldest (blue), respectively.

**
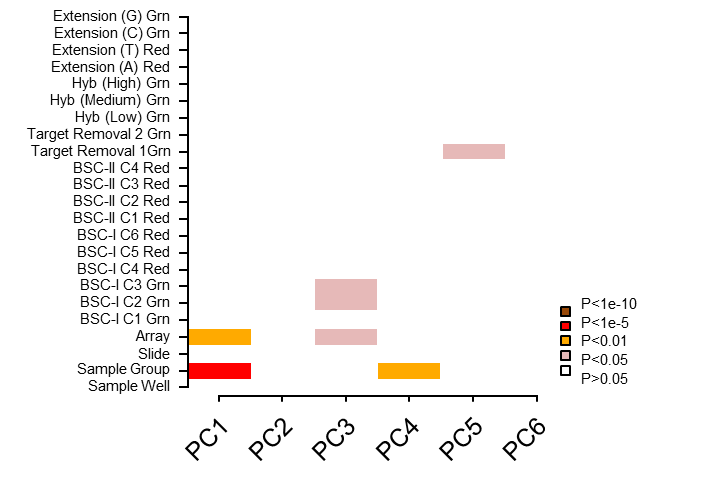
**

**Fig. S2** Singular Value Decomposition (SVD) analysis of β values. Prior to performing Principal Component Analysis (PCA) of the samples (Fig. 1B), the ChAMP Bioconductor package was used for assessing all possible sources of variation (see Experimental Procedures for details). The result shows that the statistically most significant variation comes from "Sample Group" ("young" or "old").


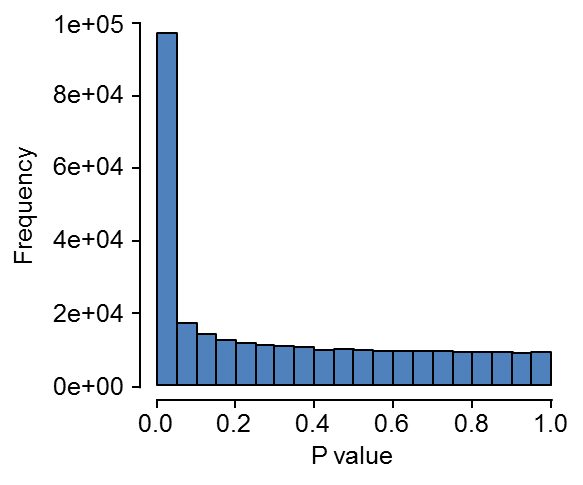


**Fig. S3** P-value frequency histogram. The graph shows an anti-conservative distribution of all unadjusted P-values. The high amount of CpGs with a P-value <0.05 suggests a negligible influence of potential confounding factors in the analysis.

**
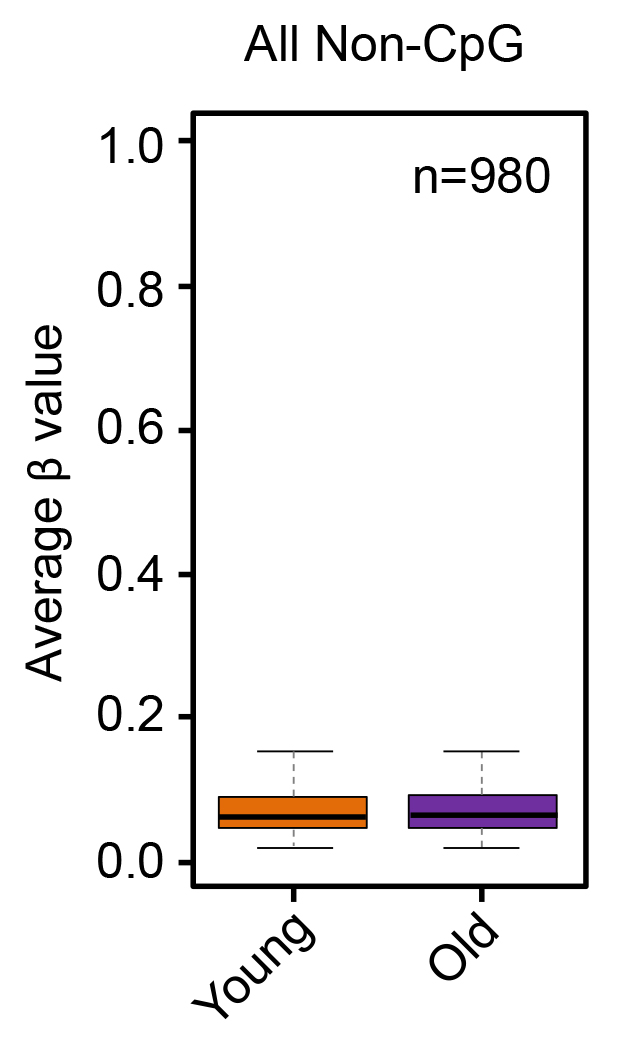
**

**Fig. S4** Analysis of non-CpG methylation. Box plots indicate the global methylation status of the 980 non-CpG probes contained on the Infinium 450k array. Levels of non-CpG methylation appeared very low, with no significant differences between the sample groups.


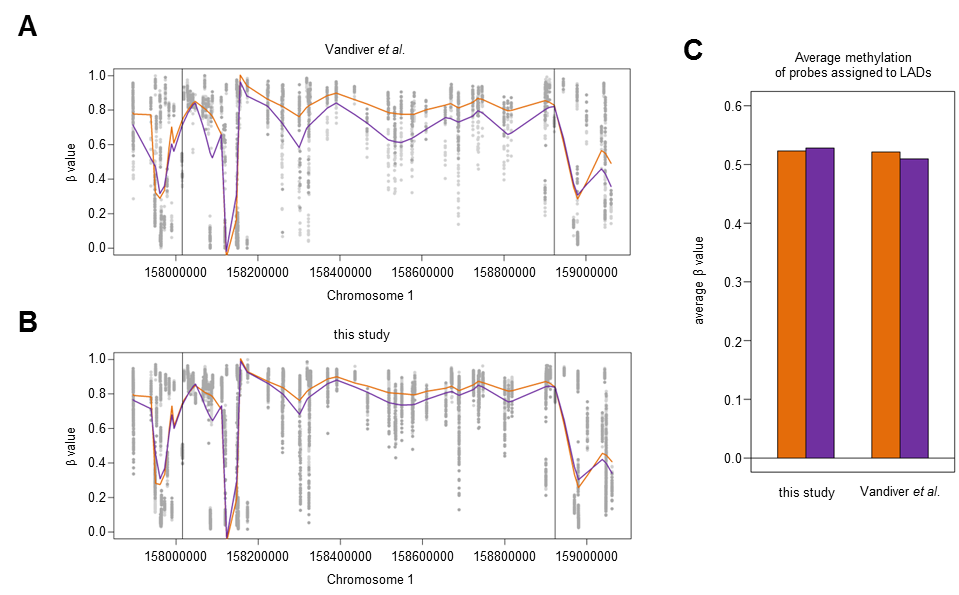


**Fig. S5** Analysis of hypomethylated blocks. (A) Methylation landscape of a prominent hypomethylated region from Vandiver *et al.* (2015). Average methylation levels in sun-protected skin samples from young individuals are shown as an orange line, average methylation levels in sun-exposed skin samples from old individuals are shown as an orange line. (B) Methylation landscape of the same region, based on the data generated in this study. Orange line: average from young samples, purple line: average from old samples. (C) Average β values of probes assigned to Lamina-associated domains (LADs). Orange bars: average methylation of young and sun-protected (Vandiver et al.) or young skin samples (this study) respectively. Purple bars: average methylation of old and sun-exposed (Vandiver et al.) or old skin samples (this study) respectively.

**
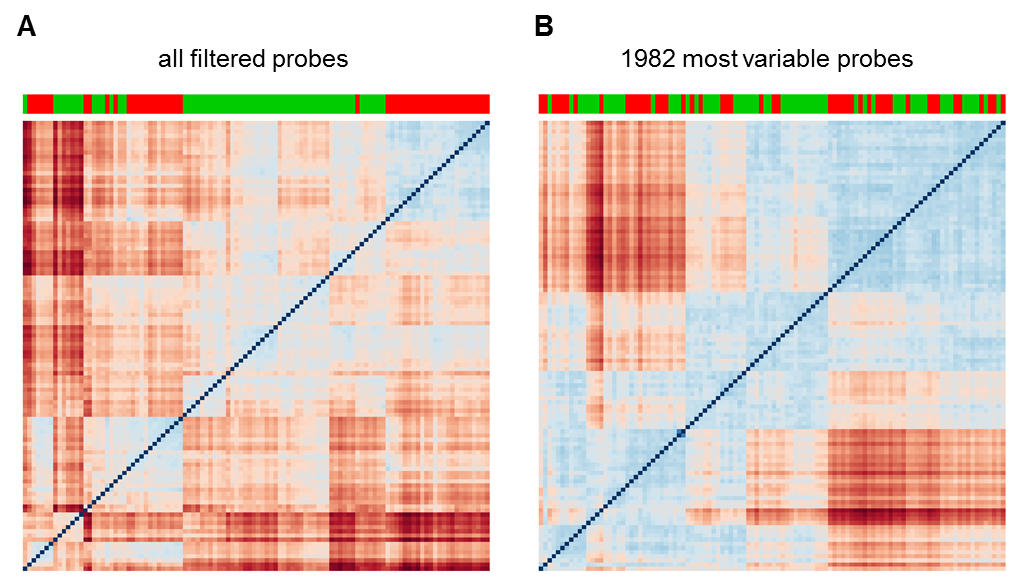
**

**Fig. S6** Cluster analysis of the complete (N=108) sample set. (A) Between-sample-distance-matrix after clustering all probes from all samples. (B) Between-sample-distance-matrix after clustering the most variable probes from all samples. Red bars on top of the panels represent the position of the 24 young and 24 old samples, green bars represent the additional 60 samples.


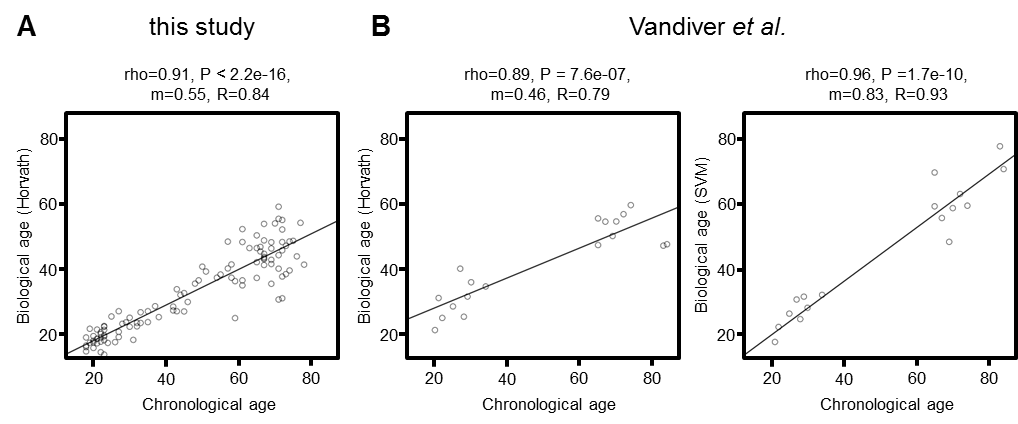


**Fig. S7** Application of age prediction methods on our data and on a published dataset. (A) The biological age was calculated as described in (Horvath, 2013) using all 450k datasets (N=108) of this study. The biological age was plotted against the associated chronological age of each sample and a linear regression curve was calculated. (B) The age prediction methods described in (Horvath, 2013) and in this study were applied to old and young sun exposed skin samples (Vandiver *et al*., 2015). Linear regression curves were calculated. For both datasets, the prediction method described by Horvath led to decreased slopes of 0.55 (this study) and 0.46 (Vandiver *et al*.) which illustrate increased absolute prediction errors of 14.5 and 12.1 years, respectively. Our method, however, showed a linear regression curve slope of 0.83 for the published dataset, thus illustrating a lesser absolute prediction error of 6.72 years.


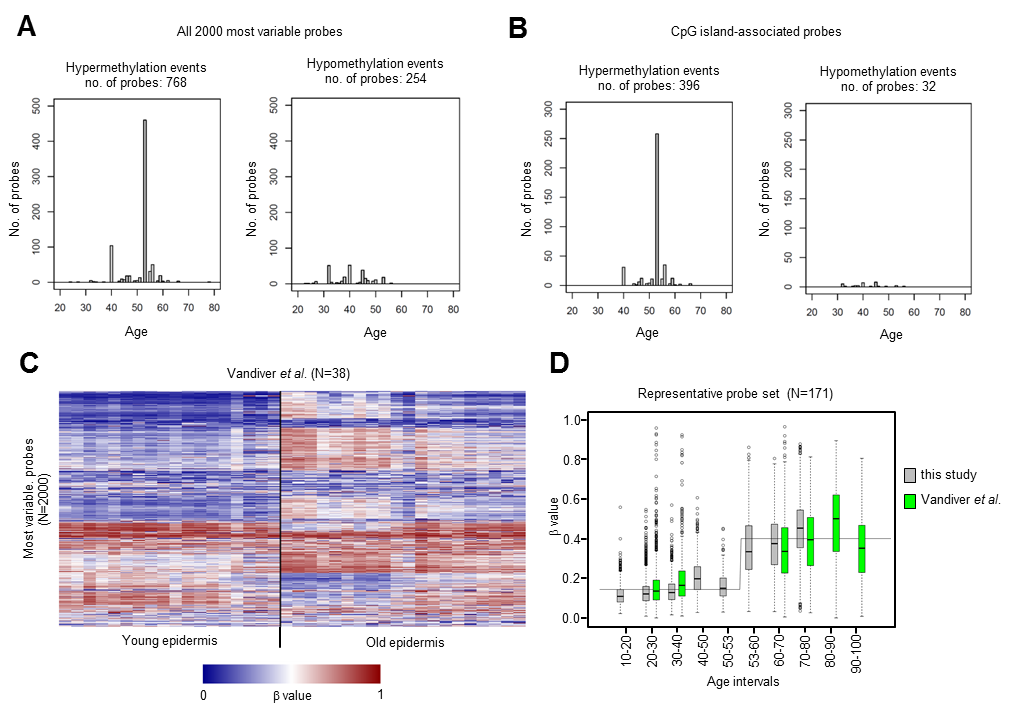


**Fig. S8** Identification of discontinuous methylation changes by recursive partitioning. (A) Hypermethylation and hypomethylation events in the most variable probes from all datasets. (B) Hypermethylation and hypomethylation events in the CpG island-associated probes from all datasets. Note that recursive partitioning identifies specific time points for subclassification that do not necessarily reflect the age distribution of the sample set. As such, the specified timepoints represent age ranges (50-60 years), rather than specific ages. (C) β value heatmap of the data generated in (Vandiver *et al.* 2015) using the 2,000 most variable probes identified in this study. The observed pattern is very similar to the pattern obtained in this study (compare to Fig. 4C). (D) Recursive partitioning analysis for a representative subset of 171 probes with similar age-related methylation changes. Grey boxes: this dataset, green boxes: (Vandiver *et al*. 2015) dataset.


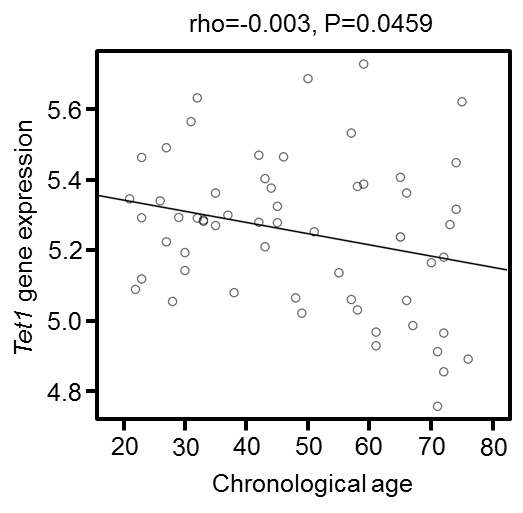


**Fig. S9** Correlation between age and the normalized gene expression level of *TET1*. The biological age was plotted against the associated *TET1* gene expression and a linear regression curve was calculated. Spearman calculation revealed a significant age-dependent decrease of *TET1* gene expression.
